# A gene drive is a gene drive: the debate over lumping or splitting definitions

**DOI:** 10.1038/s41467-023-37483-z

**Published:** 2023-03-29

**Authors:** Stephanie L. James, David A. O’Brochta, Filippo Randazzo, Omar S. Akbari

**Affiliations:** 1 Foundation for the National Institutes of Health, North Bethesda, MD 20852 USA; 2 Leverage Science, LLC, Berkeley, CA 94705 USA; 3 University of California San Diego, La Jolla, CA 92093 USA

**Keywords:** CRISPR-Cas9 genome editing, Policy

## Abstract

We address a controversy over use of the term “gene drive” to include both natural and synthetic genetic elements that promote their own transmission within a population, arguing that this broad definition is both practical and has advantages for risk analysis.

Gene drive technologies are being considered as a new approach to address a variety of currently intractable global problems, including to prevent disease transmission, reduce crop loss, and preserve biodiversity^1^. There are some outside the genetics research community who argue that wide use of the term “gene drive” to encompass selfish genetic elements found either in extant organisms (natural gene drives) or assembled in the laboratory (synthetic gene drives) will discourage the necessary scrutiny of risks that may be associated with the introduction of synthetic gene drives into free-living populations of target organisms^2,3^. Here we argue that the current definition is both scientifically sound and promotes good governance.

## The current definition of gene drive

Researchers studying selfish genetic elements have coined many terms to describe specific phenomena and systems that result in asymmetric inheritance^1^. Many of the original terms describing selfish genetic elements found in nature had mechanistic connotations, including preferential segregation, transmission ratio distortion, meiotic drive, genic meiotic drive, chromosomal meiotic drive, mitotic drive, neocentromere drive, centromere drive, B-chromosome drive, sex-chromosome drive, transposable elements, homing endonucleases, and genetic drive^4^. Today, biotechnology enables researchers to create genetic assemblies in the laboratory that mimic many of these types of natural drives. However, these synthetic, or engineered, drive systems can be purposefully designed to address particular goals, such as to reduce the prevalence of a target pest population or reduce the capacity of the target organisms to do harm. Drive mechanisms such as over-replication systems (e.g. homing), true meiotic drive systems (e.g. some toxin-antidote systems), and post-meiotic drive systems (e.g. cytoplasmic incompatibility) are currently a major focus of such research and development efforts^4^. The expression “gene drive” has emerged among applied geneticists as a useful generic term to refer to any type of transmission advantage^2^, encompassing a broad array of mechanisms utilized by both natural and synthetic selfish elements^5,6^.

## The perception that synthetic drive systems are different

There is ongoing debate about the novelty of synthetic gene drives^1,3,7,8^, particularly with regard to regulatory policies and the ability to adequately assess potential risks. In this context, it has been argued that naturally occurring selfish elements and natural drive systems should not be included in the definition of gene drive^3,9^. To a great extent, the semantics are already established in that the term gene drive is currently in wide usage among the genetics research community. However, the concerns that have been raised are largely directed not to the scientific community but to the general public’s perception of synthetic gene drive technologies and to the ability of decision-makers to adequately evaluate them. Here we propose that an artificial distinction between natural and synthetic drive systems is not warranted, and indeed can be counterproductive to the common goal of effective risk assessment and informed decision-making about new synthetic gene drive technologies.

## The scientific perspective

Research advances make it increasingly less plausible to make any meaningful distinction between natural and synthetic drives. Many synthetic gene drive systems intentionally recapitulate the mechanisms used by naturally occurring selfish elements and thus are functionally the same or similar. For example, the first efforts to create synthetic gene drives using cloning technologies involved repurposing naturally occurring transposable elements and creating chimeric assemblies with drive characteristics similar to the original transposable elements^4^. The synthetic *MEDEA* gene drive system (Maternal Effect Dominant Embryonic Arrest) simulates some ‘toxin-antidote’-type gene drive systems found widely in nature. Wolbachia (*wPip*) genes responsible for cytoplasmic incompatibility and asymmetric inheritance in *Culex pipiens* have been introduced into the genome of *Anopheles gambiae* as the potential basis for an engineered gene drive system in this mosquito^10^. Many Cas9-based synthetic gene drives configure the sequence-specific Cas9 DNA endonuclease in the same way as sequence-specific DNA-endonucleases referred to as homing endonucleases found in some pro- and eukaryotes. Not only does this configuration result in gene drive identical to that associated with homing endonucleases in nature but both gene drive systems rely on identical endogenous DNA repair mechanisms (homology directed repair) to achieve their transmission advantages. Another cogent example of the futility of any technical argument that natural and synthetic gene drives can be functionally distinguished is the recent success of a gene drive engineered to reduce the reproductive capacity of mice by using the naturally occurring *t haplotype* to spread inactivating mutations in a haplosufficient female fertility gene, thus effectively combining both natural and synthetic components within the same system^11^.

## The implications of intentionality

Another proposed rationale for distinguishing synthetic from natural gene drives is that synthetic drives are explicitly intended to serve human intentions, and therefore should not be considered as repurposed natural processes^3,9^. Here, however, it is important to remember that natural gene drives also can be used to serve human intentions. Among the earliest envisioned uses of natural gene drives employing transposable elements, meiotic drive or underdominance mechanisms was to serve human purposes by reducing transmission of vector-borne diseases^12^. For example, Craig et al. clearly recognized the potential of periodic releases of *Aedes aegypti* containing sex-ratio distorting genes in urban areas to reduce the number of females below a level required for efficient disease transmission. Similarly, Curtis proposed the use of chromosome translocations to fix desirable genes in insect pest populations. While largely unsuccessful, these early attempts to harness natural gene drive systems inspired the development of synthetic gene drive technologies as new tools for vector control. More recently, a natural gene drive has intentionally been incorporated in a population suppression strategy for mice^11^.

## The implications for good governance

Concerns also have been raised that application of the term gene drive to natural systems will result in a false sense of safety that will confuse regulatory and political discussions about synthetic gene drive technologies and undermine appropriate governance of these new biotechnology applications^3^. This presupposes that regulators will somehow equate naturalness with safety, a misconception that has been refuted elsewhere (e.g.^13^) and for which a multitude of obvious counterexamples exist (including mycotoxins, lectins, cyanogenic glycosides, aquatic biotoxins etc.), and therefore will not apply the same stringency of process. This supposition ignores the basic principles of methodologic rigor of risk analysis, which require case-by-case assessment of regulated biotechnology products whatever their composition. According to law, policy and/or regulation^14^, the testing and possibly the eventual use of synthetic gene drive systems is expected to be preceded by assessments of health risks, environmental risks, environmental and socioeconomic impacts, which will underpin decision-making at the various levels of societal organization.

Informed decision-making will be best served by taking full advantage of the science, scholarship and knowledge related to both natural and synthetic gene drives. Because synthetic gene drive systems have yet to be deployed in natural settings, empirical field data on their fate in, and impact on, the receiving environment is lacking. However, the dynamics and evolutionary fate of many natural gene drive systems have been well studied and can provide an important knowledge base for predicting the behavior of similar synthetic drive systems. In the case of homing-based synthetic gene drives, for example, except for the use of a distinct but equally sequence-specific DNA endonuclease (Cas9/sgRNA), these systems are identical to and use the same cellular machinery to achieve drive as homing endonucleases found in nature. Exclusionary semantics in this case would have the unhelpful consequences of discouraging those engaged in technology development, assessment, and governance from using some 50 years of research on natural gene drives to support discussions and decision-making. As an example of such a comparative approach, experience with a self-sustaining *Wolbachia*-mediated strategy for reducing transmission of arboviruses by *Aedes* mosquitoes is being studied as a means to predict the monitoring requirements for synthetic gene drive systems that similarly aim to reduce transmission of mosquito-borne pathogens^15^, which could contribute to more informed risk assessment and management.

## The rationale for lumping

We agree with the proposition, originating in the basic genetics research community, that understanding how synthetic drives might spread and be altered over evolutionary time will be enhanced by studying how mechanistically similar naturally occurring drive systems behave in nature^16^. Creating an artificial barrier between natural and synthetic gene drives is not only difficult to justify scientifically, but could actively undermine good governance by discouraging the consideration of important information that will inform the modeling of long-term effects, enhance risk analysis, and reduce the possibility of unintended adverse consequences of new gene drive technologies.
